# The physiological and pharmaceutical roles of MCTs and other ingredients in intravenous emulsions containing omega-3 enriched fish oil designed to mitigate cytokine release syndrome

**DOI:** 10.3389/fphar.2025.1673290

**Published:** 2025-09-22

**Authors:** David F. Driscoll, Bruce R. Bistrian

**Affiliations:** ^1^ Stable Solutions LLC, Easton, MA, United States; ^**2**^ Department of Medicine, UMASS Chan Medical School, Worcester, MA, United States; ^**3**^ Department of Medicine, Harvard Medical School, Boston, MA, United States

**Keywords:** cytokine release syndrome, CAR T cell therapy, omega-3 fatty acids, medium chain triglycerides, long chain triglycerides

## Abstract

Cytokine release syndrome (CRS) is a serious adverse effect often seen following the administration of cancer immunotherapy, particularly with chimeric antigen receptor (CAR) T cell therapy. Recently, we proposed the administration of precise amounts of the primary active ingredients found in fish oil (EPA + DHA) in combination with medium chain triglycerides (MCTs). Although there is a commercial injectable emulsion containing a refined-only fish oil, it is indicated as a nutritional supplement because it contains highly variable concentrations of the primary active ingredients (±50% of EPA + DHA). We suggested the application of a refined and enriched fish oil in order to provide the desired pharmacological doses according to the typical limits for drugs, i.e., EPA + DHA, within ±10% of the labeled amount. This tight tolerance is not achievable with “refined-only” fish oil indicated for nutrition support. The purpose of this review is to further describe the details of such a dosage form, with particular focus on other active ingredients in the proposed formulation. They play important roles in delivering a safe final product with multiple therapeutic targets for the acute systemic inflammatory response from CRS, as well as addressing chronic inflammation within the tumor microenvironment (TME).

## Introduction

Cytokine release syndrome (CRS) is a serious adverse effect that often follows the administration of cancer immunotherapy, most notably CAR T cell therapy. The acute consequences of CRS includes a hyper-inflammatory response, as well as persistent inflammation within the TME which can lead to polarization of macrophages–from suppressing tumor growth (M1 phenotype) to promoting tumor progression (M2 phenotype). Thus, prompt treatment of CRS as potentially beneficial therapy must include the initial targeting of systemic inflammation through modulation of eicosanoid metabolism recognizing its precipitous onset, while the subsequent long-term persistence of inflammation from accumulation of apoptotic cellular debris might be ameliorated as well through specialized pro-resolving mediators (SPMs) via enhanced phagocytosis and efferocytosis within the TME.

The FDA describes an active ingredient as “any component that provides pharmacological activity or other direct effect in the diagnosis, cure, mitigation, treatment, or prevention of disease…” (FDA, 2025). The National Cancer Institute’s definition is “The main ingredient in a medicine that causes the desired effect of the medicine. Some medicines contain more than one… that act in different ways in the body. Also called active pharmaceutical ingredient…” (NCI, 2025). The FDA position appears to be more broadly stated, in that it “…provides for *pharmacological activity*”, whereas the NCI definition emphasizes “…the *desired effect*”. In pointing out this difference, the FDA description appears to suggest any pharmacological effect, and that may include adverse effects, such as, for example, from drugs with a narrow therapeutic index. Thus, our approach in this review is to point out the other “active” ingredients included in the formulation that may favorably enhance their pharmacological and pharmaceutical impact on the final infusion.

## Medium chain triglycerides (8:0, 50%–80%; 10:0, 20%–50%; European Pharmacopoeia or EP 0868)

The MCTs included in the fish oil/medium chain triglycerides (FO/MCT) formulation are active ingredients that improve the metabolic utilization of enriched fish oil triglycerides, as well as the pharmaceutical quality, e.g., stability, of the emulsion. From a clinical perspective, MCTs facilitate the plasma clearance of very long chain (≥20 carbons) omega-3 fatty acid triglycerides. Second, from a pharmaceutical perspective, the addition of MCTs confers greater physical stability of lipid injectable emulsions over those containing exclusively long chain triglycerides (LCTs). Third, they may also improve the chemical stability of the polyunsaturated omega-3 fatty acids present in enriched fish oil triglycerides.

### Plasma clearance

Clearance of triglycerides from the bloodstream is largely accomplished via the enzyme lipoprotein lipase (LPL), and its efficacy depends upon the concentration of either medium or long chain triglycerides at the oil-(emulsifier)-water or o/w interface. The lower molecular weight, medium chain fatty acids (MCFA) are more water soluble than long chain fatty acids (LCFA), e.g., C8:0, 68 mg/mL vs. C16:0, 0.72 mg/mL, respectively; therefore, MCFA have a greater affinity/orientation for the aqueous region at the o/w interface than LCFA. There is ample clinical and experimental evidence of the favorable impact of MCTs on plasma clearance of infused triglycerides (Sato et al., 1994; Hamburger et al., 1998; Treskova et al., 1999; Hu et al., 2021). Most recently, uptake of omega-3 fatty acids in immune cells and blood clearance increased, and pro-inflammatory lipids decreased, when the FO/MCT ratio was an 80/20 mix, and the authors conclude that the combination of the two lipids support metabolic activity and exhibit anti-inflammatory effects (Isern et al., 2025).

To elucidate the mechanisms based on observations of improved plasma clearance of MCT-LCT mixtures, Hamilton et al. applied ^13^C NMR spectroscopy to determine the type of triglycerides that dominate at the o/w interface in MCT-LCT emulsion mixtures. They found that MCT displaces LCT at the o/w interface and this interfacial location fosters greater interaction with water-soluble proteins such as LPL, which helps explain its clinically significant impact with respect to plasma clearance (Hamilton et al., 1996).

As described previously, the longer the hydrocarbon chain length, the slower the rate of clearance of triglycerides from the bloodstream (Driscoll, 2023). Consequently, the metabolism (non-oxidative disposal) of long chain (16+ carbon chain fatty acids) triglycerides will have a greater proportion of the infused lipid droplets cleared by non-enzymatic mechanisms. When this occurs for example, plasma clearance is accomplished by significant uptake of fat droplets via the reticuloendothelial system (RES), particularly within the liver involving Kupffer cells, that has the potential for interference with immune functions (Driscoll, 2023).

### Physical and chemical stability of MCTs in fish oil-containing emulsions

In addition, the preferred location of MCT at the o/w interface may have further advantages with respect to pharmaceutical benefits from the “exterior coat” over the fish oil or FO-based LCT droplets. For example, MCTs may also improve physical stability of the emulsion in an otherwise thermodynamically unstable oil-in-water injectable dispersion. Unlike other pharmaceutical dosage forms with expiration dates from 3–5 years, injectable emulsion shelf lives are generally between 18 and 24 months. It turns out that the same “favorable” interfacial location of the MCT fraction at the surface of the o/w interface affecting plasma clearance, also results in greater physical stability of the injectable emulsion. For example, when different emulsion mixtures were evaluated by light extinction with single-particle optical sensing (LE/SPOS), MCT demonstrated less physical stress upon the egg phospholipids emulsifier, thus exerting a stabilizing influence in these LCT/MCT mixtures (Driscoll et al., 2002).

Despite the fact that lipid injectable emulsions have been commercially available in the U.S. since 1976, initially there was no pharmacopeial guidance as to assessing physical quality and *stability. Subsequently, a proposal was made for formal adoption of 2 physical methods (Driscoll, 2004), and was followed by an extensive review of the physical profiles of commercially available lipid injectable emulsions (Driscoll, 2006). On June 1, 2007, the United States Pharmacopeia (USP) formally approved Chapter <729>, entitled “Globule Size Distribution in Lipid Injectable Emulsion”, and identified LE/SPOS as the stability-indicating assay. Consequently, all intravenous lipid emulsions for FDA approval must pass this test throughout their shelf-life.

**FIGURE 1:**
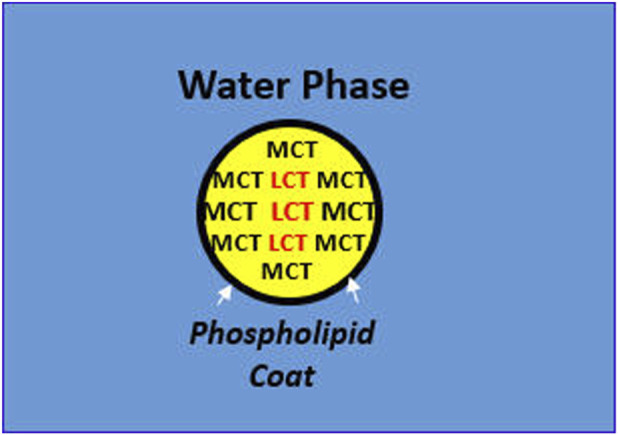
Physiological and Pharmaceutical Benefits of MCT in Emulsion Mixtures with LCT. In mixed MCT/LCT droplets, MCT displaces LCT at the oil-water interface, exerting the following benefits: Physiological Implications: Enzymatic hydrolysis of the lipid droplet by watersoluble proteins such as lipoprotein lipase is greater with MCT at the o/w interface leading to more rapid and complete metabolism. Under Pharmaceutical Implications: Interfacial tension (σ) at the o/w interface is less stressful to the phospholipid emulsifier with MCT than LCT, e.g., 8-carbon caprylic acid, σ = 8.22 dynes/cm vs. 18-carbon oleic acid, σ = 15.6 dynes/cm.

Finally, there is another potential pharmaceutical benefit, based on MCTs preferred external location alongside the emulsifier-water interface. That is, the external coating by MCTs over the FO-LCT droplets may protect the inner droplet core containing the highly polyunsaturated, omega-3 fatty acid triglycerides that are particularly susceptible to oxidation, independent of the actions of antioxidants usually present such as a-tocopherol. A summary of the key physiological and pharmaceutical benefits derived from the intravenous FO/MCT emulsion mixture are presented in Figure 1.

## Long chain essential fatty acids (LC-EFAs)

As described previously (Driscoll and Bistrian, 2025), the 3 essential fatty acids contain very long hydrocarbon chains coming from the diet that includes arachidonic acid or ARA (20:4n6), EPA (20:5n3), and DHA (22:6n3), often derived from their 18-carbon precursors, i.e., linoleic acid (18:2n6) and alpha-linolenic acid (18:3n3), respectively, via several desaturation and elongation enzymatic steps (Bistrian, 2003). Depending upon the essential fatty acid, they modulate the intensity of CRS. Furthermore, EPA and DHA are the preferred substrates over ARA for incorporation into plasma cell membranes, unless given in pharmacological amounts in the presence of ARA deficiency. As ARA is tightly controlled, once the deficiency is restored however, EPA and DHA will resume as the preferred substrates for eicosanoid metabolism, suggesting ARA incorporation is saturable (Ling et al., 1998).

Fish oil, whether as a refined-only product (EP 1912) or one that is both refined and enriched (EP 1352), does contain the essential omega-6 fatty acid ARA, which is not present when lipids are provided from plant sources (e.g., soybean oil), but only as its precursor, linoleic acid. Importantly, the therapeutic goal of enriched fish oil is to increase the concentrations of EPA and DHA, and for commercial production molecular distillation is most often applied (European Food Safety Authority, 2010). Consequently, as the concentrations of the very long-chain omega-3 fatty acids increase, the lower chain fatty acids are reduced in concentration. For example, refined-only natural fish oil contains up to about 30% of omega-3 fatty acids, whereas an oil that is also enriched results in an approximate doubling of the concentration of EPA and DHA, at the expense of lower carbon fatty acids, which are significantly reduced in concentration.

Recently we reviewed the potential application of an injectable emulsion containing a novel mixture of 9 parts enriched fish oil (FO) long chain triglycerides (LCT) and 1 part medium-chain triglycerides (MCT), designated as FO/MCT 9:1 (by wt.), suitable for intravenous administration in patients with CRS resulting from CAR T cell therapy (Driscoll and Bistrian, 2025). The principal active ingredients include the essential omega-3 fatty acids EPA and DHA, found in enriched fish oil that can be delivered in precise pharmacological doses to address the acute systemic inflammatory response as well as clearance of apoptotic cellular debris within the TME. The upper daily dose limit of EPA + DHA for therapeutic intent has been suggested to be 6 g/day (Bistrian, 2020).

### Effect of omega-3 enriched fish oil on CRS

The pathophysiology of CRS from CAR T cell therapy has two important consequences. The initial manifestation is a severe systemic inflammatory response that often occurs within 2 weeks of receiving intravenous administration (Ahmed et al., 2024), resulting from the binding of CAR T cells on the surface of target cells inducing the hypersecretion of cytokines (Renninger et al., 2025). As omega-3 fatty acids are the preferred substrate for eicosanoid metabolism, they produce less vasoactive pro-inflammatory mediators (e.g., 3-series prostaglandins vs. 2-series prostaglandins and 5-series leukotrienes vs. 4 series leukotrienes), thereby diminishing the intensity of the inflammatory response.

The second consequence arising from CAR T cell administration following successful *in vivo* expansion and a high tumoricidal impact is the accumulation of apoptotic cellular debris within the TME. Over time, the M1 macrophages are overwhelmed and can become polarized to M2 macrophages leading to tumor progression and relapse. Although CAR macrophages can be engineered to clear debris within the TME via phagocytosis and efferocytosis, there are issues with safety and efficacy (Driscoll and Bistrian, 2025). In contrast, omega-3 fatty acids are also the main substrate for beneficial SPMs that are effective and a safer approach to perform this function within the TME (Sulciner et al., 2018; Quinlivan et al., 2024).

## Long chain saturated fatty acids (LC-SFAs)

Long chain saturated fatty acids have been shown to be pro-inflammatory by serving as a ligand with toll-like receptors to initiate an immune response, occurring with even-numbered chain LC-SFAs such as palmitic acid (16:0), a property not shared by odd-chain LC-SFAs such as pentadecanoic acid (15:0) (Lee et al., 2004; Rocha et al., 2016; Nicholas et al., 2017). The clinical consequences of the pro-inflammatory actions from LC-SFAs in the diet appear to be associated with long-term, cardiometabolic diseases (Li et al., 2022). There are no data regarding specific acute effects of intravenous administration of palmitic acid from lipid injectable emulsions *per se*, which is complicated to differentiate given the concentrations of other pro-inflammatory components present in these formulations such as omega-6 fatty acids, oxidation products and phytosterols (Driscoll, 2023).

Nevertheless, if possible, it would seem desirable to limit the concentrations of pro-inflammatory mediators in lipid injectable emulsions. A comparison of selected fatty acids is therefore made between the currently available 100%, refined-only fish oil emulsion, indicated for nutritional purposes, and the proposed fish oil injectable emulsion indicated for drug therapy, which is both refined and enriched. As a consequence of the enrichment process (molecular distillation) applied to fish oil to increase the omega-3 fatty acid concentration, the levels of lower chain fatty acids are reduced. Table 1 compares the resultant differences for selected fatty acids between these different fish oils.

**TABLE 1:** Refined^a^ vs. refined and enriched^b^ fish oil of selected fatty acids.

Fatty acid	A. Refined-only fish oil^c^ ^,^ ^d^	B. Refined and enriched fish oil^c^ ^,^ ^e^	B vs. A
Saturated fatty acids			
Palmitic acid (16:0)	11.7	2.34	↓ 80%
Stearic acid (18:0)	4.4	2.83	↓ 36%
Unsaturated fatty acids			
Linoleic acid (18:2n6)	4.4	0.83	↓ 81%
α-Linolenic acid (18:3n3)	1.8	0.41	↓ 77%
Arachidonic acid (20:4n6)	2.1	2.69	↑ 28%
Eicosapentaenoic acid (20:5n3)	19.2	34.51	↑ 80%
Docosahexaenoic acid (22:6n3)	12.1	25.39	↑ 110%

^a^ Wanten and Calder, 2007 (Data provided by the manufacturer).

^b^ From Croda GmbH, certificate of analysis, batch no. 0000674901.

^c^ % by weight of total fatty acids.

^d^ European Pharmacopeia Monograph no. 1912, *Fish Oil, Rich in Omega-3, acids*.

^e^ European Pharmacopeia Monograph no. 1352, *Omega-3, acid triglycerides*.

## Intravenous infusion schedules

Due to the acute onset of CRS and associated severe adverse effects, treatment must be prompt and can only be reasonably achieved by intravenous administration (Driscoll and Bistrian, 2023). There are 3 key issues for optimizing omega-3 therapy. First, selecting the optimal dose of omega-3 fatty acids to treat CRS is essential to the success of therapy. Second, an important clinical question is whether therapy should be provided as treatment at the onset of symptoms, perhaps to allow the desired impact of CAR T cell therapy, or be given prophylactically after the CAR T cell infusion is completed in all patients to avoid or limit CRS. Finally, the infusion rate of the lipid injectable emulsion must be carefully titrated in order to avoid significant complications from fat overload syndrome, which can interfere with immune function (Driscoll, 2017). As well, the question of whether a “priming” dose should be given before initiating continuous intravenous infusion should be considered.

### Selecting the optimal dose

As described previously (Driscoll and Bistrian, 2024), and after assessment of 2 studies in critically ill patients involving more than 900 patients receiving EPA and DHA as part of a parenteral nutrition regimen (Heller et al., 2006; Wichmann et al., 2007), we suggested an upper dose limit for EPA and DHA of 6 g/day. Recommended dosages should be determined in a weight-based manner (g/kg/day) and perhaps even g/kg ideal body weight/day, e.g., 0.03–0.06 g/kg/day of EPA + DHA, and ideally, given over a large (log) weight range such as 10–100 kg, e.g., Low Dose: 0.3–3 g/d; High Dose: 0.6–6 g/day (Driscoll and Bistrian, 2025). In selecting the suggested doses, several patient-related factors considered to be important in patients receiving CAR T cell therapy, are also applicable in determining the optimal dose regimen of omega-3 fatty acid therapy for CRS, such as age, tumor burden and co-morbidities, as well as therapy-related factors such as CAR T type and dose (Renninger et al., 2025). Table 2 presents a proposal for 24-h continuous intravenous infusion, now spanning 3 different doses (0.015–0.03–0.06 g/kg/day) in an effort to identify the ideal dosing range for the treatment of CRS.

**TABLE 2:** Proposed continuous infusion schedules.

Weight (kg)	Proposed dosing range (EPA + DHA)^a^		
	0.015 g/kg/day	0.03 g/kg/day	0.06 g/kg/day
10	0.15	0.30	0.60
20	0.30	0.60	1.2
30	0.45	0.90	1.8
40	0.60	1.20	2.4
50	0.75	1.50	3.0
60	0.90	1.80	3.6
70	1.05	2.10	4.2
80	1.20	2.40	4.8
90	1.35	2.70	5.4
100	1.50	3.0	6.0
EPA + DHA Infusion Rate^b^ (g/kg/hour)	0.000625	0.00125	0.0025
Total Fat Rate (FO + MCT)	0.00125	0.0025	0.005

^a^ LipOmega-3 9/1 containing 0.2 g/mL of fat, 20%w/v (0.18 g/mL of enriched fish oil providing 100 mg/mL of EPA + DHA, and 0.02 g/mL of MCT).

^b^ Upper limit of the rate of intravenous infusion of FO-containing lipid injectable emulsions must be <0.05 g/kg/h, and preferably lower as depicted above.

### Rate of administration: push and continuous infusion

The intravenous infusion rate for all lipid injectable emulsions must be carefully applied to avoid potentially clinically significant adverse reactions relating to fat overload syndrome. As described previously, increasing the carbon chain length of the relevant fatty acid triglycerides (i.e., 8 °C→10 °C→16 °C→18 °C→20 °C→22 °C), correspondingly reduces the clearance rate from plasma (Driscoll, 2023), and most likely arises from exceeding the Km (Michaelis constant) for lipoprotein lipase. In fact, in a 1994 editorial two experts reviewed the literature and determined that when the infusion rate for soybean oil injectable emulsion (mainly consisting of 18-carbon fatty acids) exceeds 0.11 g/kg/hour, significant adverse effects occurred (Klein and Miles, 1994). When the same infusion rate above was applied to a nutrition-based, refined-only fish oil injectable emulsion however, profound hypertriglyceridemia (∼500 mg/dL) was observed (Berger et al., 2013). Clearly, the upper infusion rate for fish oil injectable emulsions must be at least 50% lower (≤0.05 g/kg/hour) or even less, and hence, therapy should be guided by these infusion limits and corresponding resultant serum triglyceride levels.

Prior to starting a continuous infusion over 24 h, a starting primer dose might be considered to be given separately by I.V. push over a brief, but fixed period of time prior to continuous infusion over 24 h. Doing so, could accelerate the achievement of therapeutic levels of omega-3 fatty acids into plasma cell membranes. In a study of 12 healthy male human volunteers (average weight: 75.8 kg; age 29.3 years), the infusion of 50 mL of a fish oil-MCT lipid emulsion containing 285 mg of EPA + DHA was administered over 5 min. The calculated infusion rate of EPA + DHA was 0.045 g/kg/hour, and there was rapid cellular enrichment of plasma cell membranes (leukocyte and platelet phospholipids) within 60 min of administration (Carpentier et al., 2010).

A continuous infusion of the novel formulation should commence at a rate <0.05 g/kg/hour, and preferably much lower as described in Table 2. Thereafter, the continuous infusion rate should be followed for the duration of therapy with a controlled syringe pump, and the syringe containing the highly enriched omega-3 fatty acid triglyceride emulsion should be aseptically replaced at 12-h intervals.

### Prophylaxis vs. treatment

The goal of therapeutic intervention with EPA + DHA for CRS is two-fold: 1) reduce the intensity of the systemic inflammatory response via eicosanoid metabolism; and, 2) clear pro-inflammatory apoptotic cellular debris within the TME via SPMs. The decision between prophylaxis vs. treatment (onset of symptoms) will have to be clinically determined during application of the proposed therapy. In principle, given the rapid onset, severity and risks associated with CRS, prophylaxis might seem to be a more prudent approach. It is important however, to recognize the significance of CAR T-cell expansion and optimum tumoricidal activity to maximize therapeutic efficacy and therefore, caution is advised to avoid the potential for mitigating *in vivo* expansion. Of note, a recent study of markers or “signatures” after CAR T cell therapy and within the TME may be prognostic for clinical outcome, and may also be of benefit to guide the timing of prophylaxis and treatment for CRS with omega-3 fatty acid therapy (Hijazi et al., 2025).

## Summary

The EPA + DHA in the enriched fish oil triglycerides that is deemed suitable for intravenous administration, as described in EP 1352, is the main active ingredient in the proposed formulation to treat CRS. As such, it must meet standard pharmacopeial requirements for the drug concentration (e.g., EPA + DHA) and it must remain within ±10% of the labelled amount at each selected time interval over the shelf life of the product. In addition, issues related to the physical stability of the lipid emulsion are equally important, as outlined in USP Chapter <729> entitled “Globule size distribution in lipid injectable emulsions”.

Other active ingredients, and in particular, medium chain triglycerides, appear to have additional clinical and pharmaceutical benefits. For example, since MCTs displace LCTs at the oil-water interface, they readily interact with lipoprotein lipase facilitating more effective enzymatic clearance from the bloodstream. Pharmaceutically, they pose 2 benefits: 1) the physical stress upon the egg phospholipids emulsifier to maintain the stability of the oil-in-water dispersion will be reduced; and, 2) the external “coating” of MCTs may further protect the highly polyunsaturated omega-3 fatty acids from oxidative degradation.

Most commonly, commercial enrichment of the fish oil is achieved by molecular distillation, resulting in significant reductions in both long chain saturated (16 and 18 carbons) and unsaturated (18 carbons) fatty acids. Hence, these additional actions not only enhance the pharmaceutical quality of the formulation, but they also increase uptake of EPA and DHA into plasma cell membranes addressing the acute systemic inflammatory response via altered eicosanoid metabolism. Furthermore, EPA and DHA are the preferred fuel for the cyclooxygenase and lipoxygenase enzymes that reduce the intensity of the inflammatory response, as well as being the key substrates for production of SPMs. Effective intravenous delivery of precise pharmacological amounts of EPA + DHA addresses the acute onset of systemic inflammation from CRS, as well as reducing the risk of cancer progression from persistent inflammation of uncleared apoptotic cellular debris within the TME.
